# Safety and effectiveness evaluation of a domestic peritoneal dialysis fluid packed in non-PVC bags: study protocol for a randomized controlled trial

**DOI:** 10.1186/s13063-015-1131-1

**Published:** 2015-12-29

**Authors:** Jianhui Zhou, Xueying Cao, Hongli Lin, Zhaohui Ni, Yani He, Menghua Chen, Hongguang Zheng, Xiangmei Chen

**Affiliations:** Department of Nephrology, Chinese PLA General Hospital, Chinese PLA Institute of Nephrology, State Key Laboratory of Kidney Diseases, National Clinical Research Center for Kidney Diseases, 28 Fuxing Road, Haidian District Beijing, 100853 China; Department of Nephrology, First Affiliated Hospital of Dalian Medical University, 222 Zhongshan Road, Zhongshan District, Dalian, Liaoning, Province 116011 China; Renal Division, Renji Hospital, Shanghai Jiao Tong University School of Medicine, 145 Shandongzhong Road, Huangpu District Shanghai, 200127 China; Department of Nephrology, Daping Hospital, Third Military Medical University, 10 Changjiangzhilu Daping, Yuzhong District Chongqing, 400042 China; Department of Nephrology, General Hospital of Ningxia Medical University, 804 Shenglinan Road, Xingqing District, Yinchuan, Ningxia, Hui Autonomous Region 750004 China; Department of Nephrology, General Hospital of Shenyang Military Command, 83 Wenhua Road, Shenhe District, Shenyang, Liaoning, Province 110016 China

**Keywords:** Comparative effectiveness research, Peritoneal dialysis, Plasticizer, Randomized controlled trial

## Abstract

**Background:**

Peritoneal dialysis is an important type of renal replacement therapy for uremic patients. In peritoneal dialysis, fluids fill in and flow out of the abdominal cavity three to five times per day. Usually, the fluid is packed in a polyvinyl chloride (PVC) bag. Safety concerns have arisen over di-(2-ethylhexyl) phthalate, which is essential in the formation of PVC materials. In 2011, the National Development and Reform Commission of China released a catalog of industrial structural adjustments, mandating the elimination of PVC bags for intravenous infusion and food containers. Although bags for peritoneal dialysis fluid were not included in the elimination list, several manufacturers began to develop new materials for fluid bags. HUAREN peritoneal dialysis fluid consists of the same electrolytes and buffer agent as in Baxter fluid, but is packed in bags that do not contain PVC. This multicenter randomized controlled trial was designed to compare peritoneal dialysis fluid packed in non-PVC-containing and PVC-containing bags. Further, the study sought to determine the proper dose of peritoneal dialysis fluid and the actual survival rates of Chinese patients undergoing peritoneal dialysis.

**Methods/Design:**

The study participants are adults undergoing continuous ambulatory peritoneal dialysis for 30 days to 6 months. All eligible patients are randomized (1:1) to peritoneal dialysis with Baxter and HUAREN dialysis fluids (initial dose, 6 l/day), with dosages adjusted according to a unified protocol. The primary outcomes are the 1-, 2-, 3-, 4-, and 5-year overall survival rates. Secondary outcome measures include technique survival rates, reductions in estimated glomerular filtration rate, nutritional status, quality of life, cardiovascular events, medical costs and drop-out rates. Safety outcome measures include adverse events, changes in vital signs and laboratory parameters, peritonitis, allergies, and quality of products.

**Discussion:**

This study is the first to evaluate the long-term safety and effectiveness of a non-PVC packed peritoneal dialysis fluid. The effects of plasticizer on patient long-term survival will be determined. The characteristics of Chinese patients undergoing peritoneal dialysis will be determined, including proper dose, technique survival rates, patient survival rates, and medical costs.

**Trial registration:**

Clinicaltrials.gov NCT01779557.

**Electronic supplementary material:**

The online version of this article (doi:10.1186/s13063-015-1131-1) contains supplementary material, which is available to authorized users.

## Background

Peritoneal dialysis is a major type of renal replacement therapy. Worldwide, up to about 200,000 patients, or about 12 % of all patients with end-stage renal disease, currently undergo peritoneal dialysis. This therapy involves filling of the abdominal cavity and drainage of peritoneal dialysis fluids three to five times per day, with each patient requiring 6 to 10 liters of peritoneal dialysis fluid per day. *Dianeal*® (Baxter Inc.) is the most widely used commercially available peritoneal dialysis fluid in China and has been used for many years. The dialysis fluid is packed in a bag made of polyvinyl chloride (PVC). The plasticizer most frequently added to PVC to improve its flexibility is di-(2-ethylhexyl) phthalate (DEHP). However, DEHP can be released by PVC materials because it is not chemically bonded to the polymer. Moreover, DEHP can be incorporated into the human body and be detected in body fluids and tissues. It has been shown that DEHP is toxic to the liver, testis, and other organs [1, 2], and that its major metabolite, mono-(2-ethylhexyl) phthalate, is even more toxic than DEHP. Use of this plasticizer has aroused great concern regarding the safety of PVC materials [3, 4]. Many daily utensils contain plasticizer, which might be harmful to human health [5–10]. Medical tubing and bags made of PVC, such as bloodlines for hemodialysis and bags for peritoneal dialysis fluids, might expose patients to considerable amounts of plasticizer and its hydrolytic metabolites. The safety of plasticizers in medical materials has been evaluated by the USA, Japan, and the European Union for more than 10 years. In 2011, the Chinese National Development and Reform Commission released a catalog of industrial structural adjustments [11], mandating the elimination of PVC bags for infusion and food containers. Although bags for peritoneal dialysis fluids were not included on that list, the presence of higher concentrations of DEHP in patients receiving continuous ambulatory peritoneal dialysis [12] has led manufacturers to seek new materials for peritoneal dialysis bags. HUAREN peritoneal dialysis fluid is a non-PVC packed Chinese product that contains the same electrolytes and buffering agent as Baxter fluid. Several studies have investigated non-PVC peritoneal dialysis fluid products (e.g., *bicaVera*®, *balance*®) [13, 14]. However, because the packaging material is the only difference between HUAREN and Baxter peritoneal dialysis fluids, a study comparing the two should clearly show the effects of plasticizer.

The targeted small solute clearance for Asian patients is *Kt*/*V* ≥ 1.7 [15], although other targets have been suggested [16–19]. The proper dose required to attain this goal may differ among populations, owing to differences in race, ethnicity, and food consumption. A dosage of 8 l/day is often used in Western populations, whereas the dosage for Asians is usually 6 l/day. Our previous research showed that an initial dose of 6 l/day could provide adequate peritoneal dialysis for 48 weeks [20], but the proper dosage afterwards remains unclear. The proper dosage for Chinese patients receiving continuous ambulatory peritoneal dialysis is unclear, as are their mid- to long-term survival rates. This study includes a protocol for adjusting dosage based on each patient’s medical status. With a 5-year follow-up period, the ‘proper’ dosage and ‘real’ situations of Chinese patients receiving continuous ambulatory peritoneal dialysis can be determined.

## Methods/Design

### Study design

**Fig. 1:**
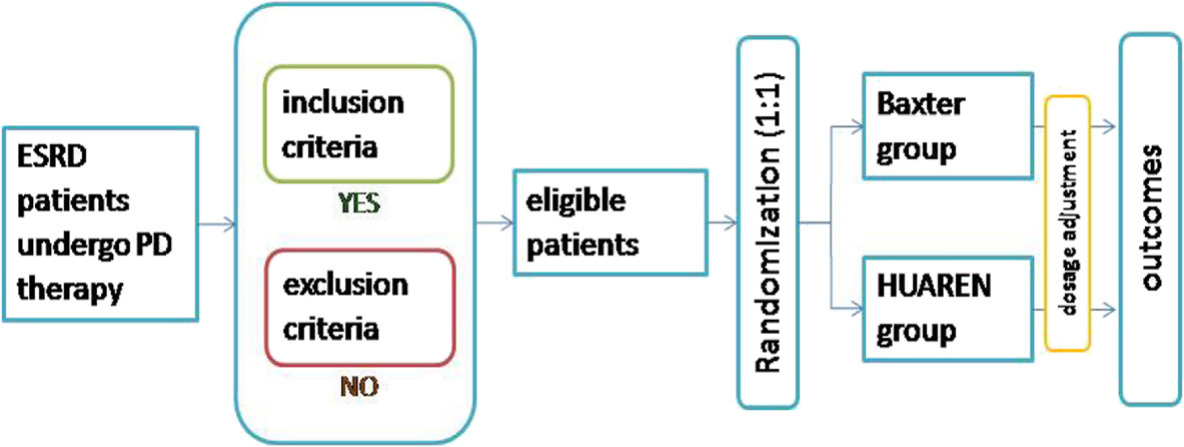
Study design. This is a randomized controlled trial. The eligible patients will be randomly allocated to two groups, Baxter group or HUAREN group. Patients will use Baxter or HUAREN peritoneal dialysis fluid for 5 years. The outcomes will be analyzed and compared. ESRD, end-stage renal disease; PD, peritoneal dialysis

This is a prospective, multicenter, randomized controlled trial with non-inferiority design. Patients will be randomized to HUAREN or Baxter peritoneal dialysis fluid (Fig. 1).

### Patients

Male and female patients aged ≥18 years diagnosed with end-stage renal disease who undergo continuous ambulatory peritoneal dialysis for 30 to 180 days are included. Patients diagnosed with diabetic kidney disease have a residual glomerular filtration rate of 3 to 15 ml/min [21], whereas patients with other renal diseases have a residual glomerular filtration rate of 3 to 10 ml/min.

All patients must provide written informed consent before undergoing any study-related procedures.

Patients are excluded if: they have acute renal failure; are candidates for kidney transplantation; are undergoing hemodialysis; have an exit site infection, tunnel infection or peritonitis according to Peritoneal Dialysis Standard Operating Procedure [22]; are anti-HIV positive; are allergic to any component of dialysis fluids; have a systemic infection, malignancy, liver cirrhosis, severe congestive heart failure, anemia (hemoglobin <80 g/l), malnutrition (albumin <28 g/l), refractory hypertension, high peritoneal transportation (*D*/*P*_Cr_ > 0.81); or are pregnant or lactating. Patients with poor compliance or a history of alcoholism or drug abuse are also excluded.

Patients are being recruited from 50 peritoneal dialysis centers throughout China. The study protocol was approved by the ethics committee of each center (Additional file 1) and is conducted according to the Declaration of Helsinki [23] and the Good Clinical Practices guidelines of the China Food and Drug Administration [24]. The study has been registered at www.clinicaltrials.gov, number NCT01779557.

### Randomization

Patients are centrally randomized 1:1 to HUAREN or Baxter peritoneal dialysis fluid, using a randomization chart generated by a biometrician blinded to clinical treatment. Physicians and nurses are also blinded to the randomization process, and each patient can be randomized only once. All procedures are under the supervision of an independent clinical research organization.

### Interventions

#### HUAREN group

Patients randomized to the HUAREN group will receive HUAREN dialysis fluid, a medical product that has been available in China for years. All the ingredients of HUAREN peritoneal dialysis fluid are identical to those of Baxter peritoneal dialysis fluid except for the packaging material, in that HUAREN peritoneal dialysis fluids are packed in bags made of non-PVC materials.

#### Baxter group

Patients randomized to the Baxter group will receive Baxter *Dianeal* dialysis fluid. This product contains 1.5 % dextrose as an osmotic agent and 40 mmol/l lactate as a buffering agent. The pH of this dialysis fluid is 5.5, and total osmotic pressure is 346 mOsm/kg. Other ingredients include 1.75 mmol/l Ca^2+^, 0.25 mmol/l Mg^2+^, 132 mmol/l Na^+^, and 96 mmol/l Cl^−^. Baxter dialysis fluids are packed in bags made of PVC materials.

During the screening period, all patients receive HUAREN dialysis fluid. After randomization, patients receive the allocated dialysis fluid.

#### Protocol for dosage adjustment

The initial dose in the two groups is 6 l/day, allowing three fluid exchanges. If *Kt*/*V*_total_ < 1.7, the dose is increased to 8 l/day, allowing four fluid exchanges. If *Kt*/*V*_total_ is ≥ 1.7, but with volume overload (e.g., heart failure, serous cavity effusion, moderate or severe edema), salt and water intake is restricted and diuresis starts. If this is insufficient, dextrose concentration is increased to 2.5 % or 4.25 %, depending on clinical demands. If a patient’s condition does not improve after administration of >6 l of fluid containing 2.5 % dextrose or >2 l of fluid containing 4.25 % dextrose, the dosage is increased to 8 l/day.

If *Kt*/*V*_total_ ≥ 1.7 and the patient is euvolemic, but with uremic manifestations, such as nausea, vomiting, or pruritus, the dose is increased from 6 l/day to 8 l/day.

**Fig. 2:**
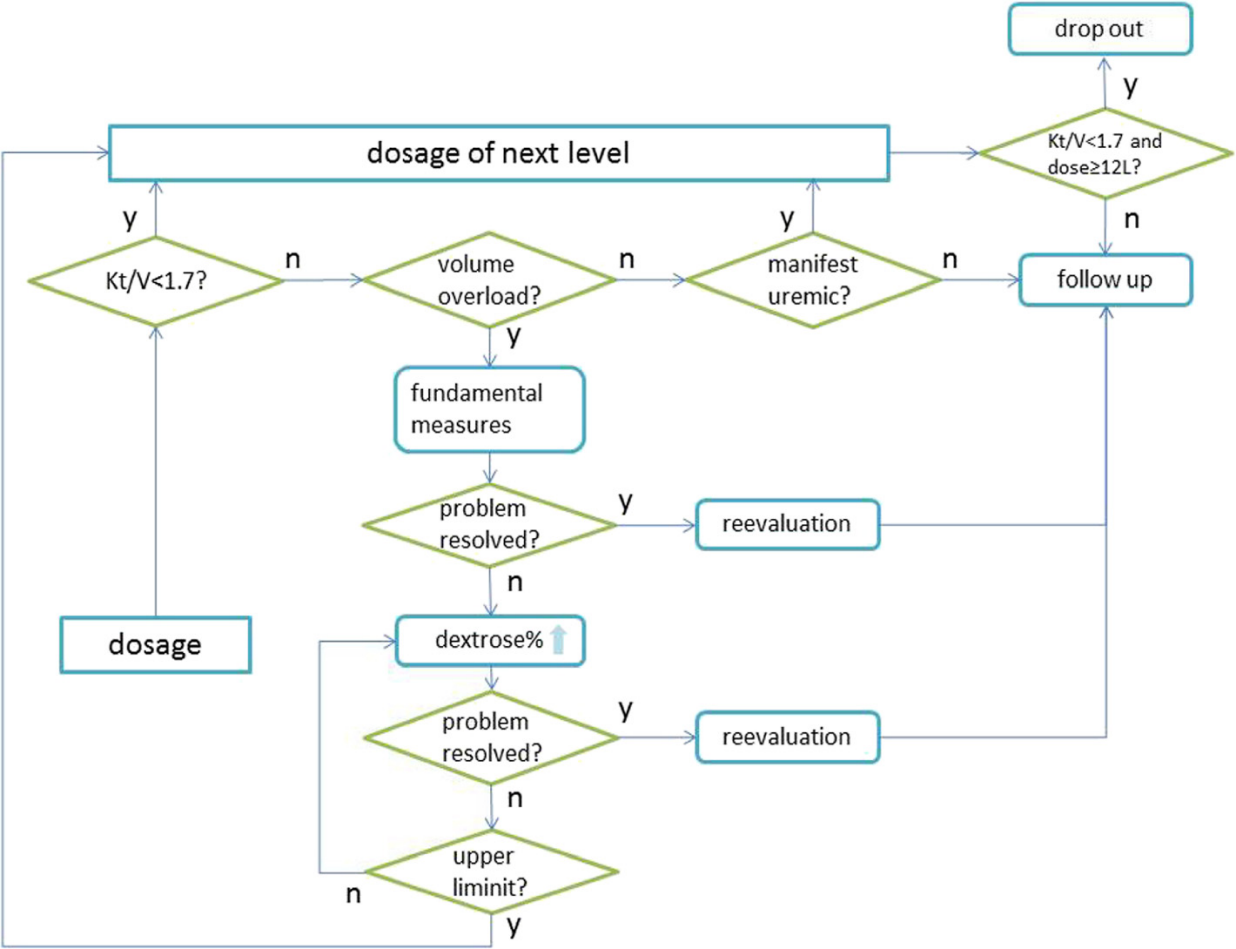
Dosage adjustment protocol. The initial dosage is 6 l/day; then the solute clearance, water removal, and clinical manifestation are evaluated. If *Kt*/*V* < 1.7, dosage should be increased. If there is a volume overload, salt and water intake, diuretics, and dextrose concentration should be modified before changing dosage. When the dosage is ≥ 12 l/day and *Kt*/*V* < 1.7, the treatment adaptation fails. Physicians should be flexible in choosing optimal therapies to protect patients from life-threatening circumstances. The *Kt*/*V* and creatinine clearance rate should be re-evaluated 2 weeks after each patient’s situation stabilizes

Dosage changes from 8 l/day to 10 l/day should follow the same principle and procedure (Fig. 2).

### Outcomes

The primary outcome measure is patient survival rates at 1, 2, 3, 4, and 5 years. The secondary outcome measures include peritoneal dialysis technique survival rates of 1, 2, 3, 4, and 5 years; estimated decline in glomerular filtration rate, nutritional status (represented as serum albumin and Subjective Global Assessment Score [25]), quality of life (described by the 36-Item Short-Form Health Survey [26]), cardiovascular events (cardiac sudden death, myocardial infarction, severe arrhythmia, carotid plaque, heart failure, cardiovascular intervention, coronary heart disease without myocardial infarction, stroke or transient ischemic attack, peripheral artery disease), increments of dialysis dosage from baseline to the end, and drop-out rates. Medical costs are also recorded.

Safety evaluations include adverse events and severe adverse events, marked changes of vital signs and laboratory test results, peritonitis (infectious or chemical), allergy to ingredients of peritoneal dialysis fluids and materials of bags and tubing, and quality of dialysis product. Evaluation of product quality includes the transparency of fluid bags, the tightness of connections, and the legibility of labels and texts on packaging.

### Sample size

The sample size is based on both Chinese facilities containing large numbers of patients [27–31] and the Chinese National Renal Data System [32], consisting of smaller units. We have estimated that the mean 5-year survival rate of patients using Baxter dialysis fluid is about 50 %, with a hazard ratio of 1.31. If the recruitment lasts 2 years and the observation 5 years, with *β* = 0.8, a one-sided *α* for the *t* test = 0.025, and 15 % censored data in each group, 310 patients per group will be needed. With a 20 % drop-out rate, 750 patients should be enrolled in this study.

### Statistics

The primary analysis population is the per-protocol set, consisting of randomized patients with baseline data and data on primary outcome measures, as well as good compliance. This population includes patients who quit the study because of lack of drug effectiveness. The secondary analysis population is the full analysis set according to the intention-to-treat principle; this population includes all randomized patients who receive therapy at least once, regardless of whether they adhere to the protocol or provide a complete dataset. Patients who never receive therapy are excluded. Patients with missing data are included, with adjacent data carried forward to the missing data. The full analysis set population is consistent throughout the entire study. The safety analysis population is the safety set, which includes all patients who receive therapy at least once.

Patient survival rates are analyzed by constructing a Cox regression model that includes multiple variables, such as baseline glomerular filtration rate, severity of disease, age, and treatment group. A non-inferiority test is determined by computing the 95 % confidence interval of the hazard ratio. The same method is used to analyze technique survival rates. Decline in estimated glomerular filtration rate is analyzed by the Wilcoxon rank-sum test. Nutritional status and quality-of-life score are analyzed by analysis of covariance. Rates of cardiovascular events and patient drop-out are analyzed by the Cochran–Mantel–Haenszel *χ*^2^ test. Increments of dialysis dosage from baseline to the end of treatment are analyzed by constructing a mixed model. Medical costs are compared by *t* test or analysis of variance. Adverse events and severe adverse events are categorized according to terminology in the Medical Dictionary for Regulatory Activities. Adverse events, severe adverse events, peritonitis, and allergies are compared by the *χ*^2^ test or Fisher’s exact test. Product quality is evaluated by nonparametric tests.

## Discussion

Peritoneal dialysis has become one of the most important renal replacement therapies. Because peritoneal dialysis is equivalent to or better than hemodialysis in the early stage [33], as well as its other advantages, including lower cost, greater convenience, better preservation of residual renal function, and greater ability to accommodate more patients with end-stage renal disease because of its lower infrastructure requirements [34], the use of peritoneal dialysis is continuously expanding worldwide, especially in developing countries [35]. At present, about 12 % of patients with end-stage renal disease worldwide are treated with peritoneal dialysis. In China, about 16 % of patients with end-stage renal disease, or about 50,000 individuals, undergo peritoneal dialysis, and the number is increasing dramatically [32]. A combination of increasing affluence, urbanization, changing lifestyles, and high rates of diabetes have all been associated with a dramatic increase in the numbers of patients who require dialysis [36].

According to data from the National Bureau of Statistics of China, the population of mainland China at the end of 2013 was 1,360,720,000 individuals [37], and the prevalence of chronic kidney disease was about 10.8 % [38]. Thus, over 140 million people in China are estimated to have chronic kidney disease. Diabetes, a major cause of end-stage renal disease, has a prevalence of 11.6 %, affecting about 120 million persons [39]. Hypertension, another major cause of end-stage renal disease, has a much higher prevalence, of 26.6 %, in Chinese adults, affecting an estimated 360 million people [40], and a newly published survey found that the prevalence was much higher [41]. The increasing numbers of patients with these causes of end-stage renal disease make chronic kidney disease a public health problem. Because many patients in China cannot afford treatment for end-stage renal disease, the Chinese government has launched a series of new policies for the use of peritoneal dialysis and extension of medical insurance coverage. Efforts are underway to improve peritoneal dialysis techniques and products to treat more uremic patients.

In China, the appropriate peritoneal dialysis dosage remains unclear. Western patients are often treated with 8 l/day, whereas 6 l/day is believed sufficient for incident peritoneal dialysis in Asian patients. Chinese patients differ ethnically from patients in Western countries. Although Chinese patients are similar ethnically to patients in Hong Kong and Japan, their economic status, which is important for peritoneal dialysis use, is quite different. Our previous study showed that 6 l/day was sufficient for adequate peritoneal dialysis in Chinese patients, although a 48-week follow-up showed continuous decline in *Kt*/*V* and creatinine clearance rate [20]. Further decreases in *Kt*/*V* and creatinine clearance rate would necessitate increases in peritoneal dialysis dosage. In this study, the observation period is 5 years. Thus, dosage could not be fixed at 6 or 8 l/day; rather, it would have to be changed according to clinical situations. The trial described here would therefore be a study of the effectiveness, not the efficacy, of peritoneal dialysis [42]. The dosage adjustment protocol in this study is almost the same as that in routine clinical circumstances.

This study compares two types of peritoneal dialysis fluid, one packed in PVC and the other in non-PVC materials. It is known that PVC has deleterious effects on human health, owing to the added plasticizer (i.e., DEHP), although the significance of its toxicity may be exaggerated [43, 44], Moreover, an as-yet-unknown toxicity may manifest during long-term usage. The toxicity of the plasticizer has been confirmed [45, 46], limiting the usage in some countries of PVC materials in medical devices and food and drug utensils [47]. The Chinese National Development and Reform Committee released a catalog of industrial structural adjustments in 2011, although peritoneal dialysis fluids were excluded from the list [48]. However, use of the plasticizer remains a public and medical concern. This is not the first trial to compare PVC and non-PVC materials in peritoneal dialysis, but it is the first long-term head-to-head comparison of peritoneal dialysis fluids packed in PVC and non-PVC materials. Studies have compared the traditional PVC packed dialysis fluid *Dianeal*® and non-PVC packed peritoneal dialysis fluids, but these fluids have many differences other than their packaging, including pH and buffering agent. The two types of peritoneal dialysis fluid in this trial have the same pH, buffer, and osmotic agents, with only the packaging material differing, in that *Dianeal*® is packed in PVC and HUAREN dialysis fluid in non-PVC bags.

This study may also reveal some of the disadvantages of non-PVC material. For example, this material may be stiff and fragile, and may be prone to leakage when used for peritoneal dialysis, resulting in peritonitis. The PVC packaging has better elasticity, flexibility, and durability, making it useful as a liquid container. In a large country, such as China, peritoneal dialysis fluids travel a great distance to reach patients’ homes. Climate, temperature, and physical abrasion all challenge the packaging of dialysis fluids. Transportation of peritoneal dialysis fluids to patients in the far north of China is especially long and difficult and may have deleterious effects if the packaging is not durable. Thus, it remains unclear whether the use of non-PVC packed peritoneal dialysis fluids is feasible in China.

This study also involves some social and economic aspects, including patient quality of life, the quality of dialysis products, and medical costs. This study reflects real-life situations, making it more relevant to setting policy and evaluating the effects of treatment. This study, a type of comparative effectiveness research, may provide useful information about Chinese peritoneal dialysis patients and suggest reasonable policies to the Chinese government. China is undergoing comprehensive reform, including healthcare reform. Three years ago, former premier Wen Jiabao announced that the Chinese government would fully introduce social insurance against uremia. Mr Chen Zhu, former leader of the Chinese Ministry of Health, put forward a series of healthcare policies to improve uremia care and to use domestic dialysis products [49]. HUAREN peritoneal dialysis fluid is a Chinese domestic non-PVC packed dialysis product that has been on the market for years. Thus, the results of this non-inferiority trial may enable the government to make a decision. If the non-PVC material is not inferior to PVC material, HUAREN should be further developed. If, however, the non-PVC material is inferior to PVC, the legal situation of PVC should be clarified. Regardless of the results, additional well-designed randomized controlled trials and cohort studies will be needed to obtain additional high-level evidence about Chinese peritoneal dialysis products. ‘To resolve our own problems with domestic products’ is not only a slogan but also a useful method for developing peritoneal dialysis in a large developing country like China. Moreover, it may provide experience that can be used by other developing countries. It should be noted that we exclude patients who are keen to receive a transplant quickly, because these patients are usually not the healthiest, and the transplantation incidence is often not in accordance with epidemiological rules. This may cause some bias, which we should pay attention to when translating the results of the study to other peritoneal dialysis populations.

## Trial status

This study is currently recruiting participants.

## Additional file

Additional file 1:
**The ethical bodies that approved the study in the various centers.** This file includes the names of all the ethical bodies who approved the study protocol in every center participating in this clinical trial. (DOC 34 kb)

## Abbreviations

DEHP: di-(2-ethylhexyl) phthalate
PVC: polyvinyl chloride
