# Supplementary material for: Safety and effectiveness evaluation of a domestic peritoneal dialysis fluid packed in non-PVC bags: study protocol for a randomized controlled trial
Source: Trials. 2015 Dec 29;16:592. doi: 10.1186/s13063-015-1131-1 (PMC4693427; doi:10.1186/s13063-015-1131-1)
Supplement: Additional file 1: — The ethical bodies that approved the study in the various centers. This file includes the names of all the ethical bodies who approved the study protocol in every center participating in this clinical trial. (DOC 34 kb) [file 13063_2015_1131_MOESM1_ESM.doc]

**The ethical bodies that approved the study in the various centers**

1. Ethics committee of the Chinese People's Liberation Army General Hospital
2. Drug clinical research IRB of Peking University Third Hospital
3. Ethics committee of China-Japan Friendship Hospital
4. Ethic committee of Capital Medical University affiliated Beijing Friendship Hospital
5. Ethics Committee of 306 Military Hospital of China
6. Ethics Committee of First Hospital of Jilin University
7. Ethics Committee of Second Hospital of Jilin University
8. Ethic Committee of Shengjing Hospital of China Medical University
9. The ethic committee of North Hospital of Shenyang
10. Medical Ethics Committee, the First Affiliated Hospital of Dalian Medical University
11. Medical Ethics Committee, the Second Affiliated Hospital of Dalian Medical University
12. Ethics Committee of the First Hospital of Shanxi Medical University
13. Ethics Committee of the Second Hospital of Shanxi Medical University
14. IEC, Henan Provincial People’s Hospital
15. Clinical Trial Ethics Committee/IRB of Qingdao Municipal Hospital
16. Medical Ethics Committee of the Affiliated Hospital of Medical College of Qingdao University
17. Ethics Committee of Zhongshan Hospital, Fudan University
18. Ethics Committee of Huashan Hospital, Fudan University
19. Ethic committee of Renji Hospital affiliated to Shanghai Jiaotong University School of Medicine
20. IRB/ Ethics Committee of Shanghai First People's Hospital
21. Ethics Committee of Changzheng Hospital
22. Institutional Review Boards of the First Affiliated Hospital of Nanjing Medical University, Jiangsu Province Hospital
23. Ethics Committee of Nanjing Drum Tower Hospital
24. Ethics Committee of the Second Affiliated Hospital of Soochow University
25. Ethics Committee of the First Affiliated Hospital of Zhejiang University School of Medicine
26. Ethics Committee of the Second Affiliated Hospital of Zhejiang University School of Medicine
27. Ethics Committee of Zhejiang Provincial People' s Hospital
28. Ethics Committee of the First Affiliated Hospital of Anhui Medical University
29. Ethics Committee of the Second Affiliated Hospital of Anhui Medical University
30. Ethics Committee of the First Affiliated Hospital of Fujian Medical University
31. Research Ethics Committee of Guangdong General Hospital, Guangdong Academy of Medical Sciences
32. Ethics Committee of Affiliated Hospital of Guangdong Medical College
33. Ethics Committee of Renmin Hospital of Wuhan University
34. Ethics Committee of Second Xiangya Hospital of Central South University
35. Ethics Committee of the First Affiliated hospital of Guangxi Medical University
36. Ethics Committee of the Second Affiliated Hospital of Kunming Medical University
37. Ethics Committee of Daping Hospital, Third Military Medical University, Chongqing
38. Ethics Committee of West China Hospital of Sichuan University
39. Sichuan Academy of Medical Sciences & Sichuan Provincial People's Hospital Medical ethic committee
40. Ethics Committee of the First Affiliated Hospital of Xi'An Jiaotong China Hospital Sichuan University
41. Ethic Committee of Xijing Hospital, Fourth Military Medical University
42. Ethic Committee of Tangdu Hospital, Fourth Military Medical University
43. Ethic Committee of General Hospital, Ningxia University
44. Ethics Committee of First Affiliated Hospital of Xinjiang Medical University
45. Ethics Committee of Changhai Hospital
46. Ethics Committee of Ningbo No. 2 Hospital
47. Ethics Committee of the Third Hospital of Hebei Medical University.
48. Ethics Committee of Jiangxi Provincial People's Hospital
49. Ethics Committee of the First Affiliated Hospital of Baotou Medical College
50. Ethics Committee of Dalian Municipal Central Hospital
